# The predictive significance of uric acid to high density lipoprotein- cholesterol ratio and uric acid for the severity and mortality of coronavirus disease-19

**DOI:** 10.1186/s13104-024-06807-7

**Published:** 2024-09-27

**Authors:** Marzieh Rohani-Rasaf, Farideh Ghavidel, Hossein Hosseini, Maryam Teimouri

**Affiliations:** 1 Department of Epidemiology, School of Public Health, Shahroud University of Medical Sciences, Shahroud, Iran; 2 Department of Clinical Biochemistry, Faculty of Medicine, Mashhad University of Medical Sciences, Mashhad, Iran; 3 Department of Clinical Biochemistry, Faculty of Medicine, Shahroud University of Medical Sciences, Shahroud, Iran

**Keywords:** Uric acid, HDL-c, Uric acid to HDL-c ratio, UHR, COVID-19

## Abstract

**Objective:**

The non-invasive and inexpensive predictive indicators seem to be essential for the evaluation of coronavirus disease-19 (COVID-19) prognosis. Uric acid to high-density lipoprotein-cholesterol ratio (UHR) have been known as inflammatory and metabolic biomarker in some disorders. This study aimed to evaluate the usefulness of serum uric acid (UA) and UHR values on admission as prognostic indicators for the severity and mortality of COVID-19. Regression models were accomplished to assess the association between UA and UHR with the severity and mortality of COVID-19.

**Results:**

This study was performed with 424 confirmed COVID-19 patients. The mean UA and UHR values of the severe group and deceased group were statistically higher than those mild group and survivor group, respectively (*P* < 0.05). Compared to the survivor cases, deceased subjects had lower serum concentrations of HDL-c (*p* < 0.05). Multivariate logistic regression analysis showed that UHR and UA values statistically are correlated with the severity (OR = 1.20 CI:1.07–1.35, OR = 1.19 CI:1.023–1.381 respectively) and mortality (OR = 10.04 CI:1.50–67.30, OR = 10.73 CI:1.47–87.11, respectively) of COVID-19. Compared with a reference range, serum UA levels ≥ 7.3 mg/dl and a UHR value greater than 0.185 increase the risk of critical care of COVID-19 almost 2.5 and 3.5 times, respectively. In summary, our results revealed that UHR index value and serum UA levels are useful biochemical indicators for predicting the severity and mortality of COVID-19.

## Introduction

The global outbreak of coronavirus disease-19 (COVID-19) pandemic causes a wide range of clinical manifestations, from mild respiratory symptoms to pneumonia and, in more severe cases, multiple organ failure [1, 2]. Due to challenges in managing COVID-19, early diagnosis and hospitalization, effective use of intensive care services, and appropriate treatment are essential for the maximum survival of patients [3].

It has been demonstrated that the risk of severe consequences of COVID-19 increases in patients suffering from some comorbidities such as metabolic syndrome, type 2 diabetes mellitus (T2DM), cardiovascular disease (CVD), and metabolic fatty liver [4]. It is worth noting that, the loss of metabolic health seems to be the common denominator for all these comorbidities. Therefore, in addition to clinical assessment, using non-invasive and low-cost laboratory markers that show the patients’ metabolic status can predict the COVID-19 prognosis and improve the patient care components [3]. Studies revealed several laboratory markers and indices such as C-reactive protein (CRP), Lactate dehydrogenase (LDH), high-sensitivity cardiac troponin I (hs-cTnI) [5], lipid ratios, triglyceride to glucose index (TyG) [6] can predict the prognosis of COVID-19.

It has also been demonstrated that uric acid (UA), the final product of purine catabolism, is closely related to the activation of the immune system and the elimination of reactive oxygen species (ROS) [7]. However, some studies have stated that hyperuricemia is a causative factor in the development of insulin resistance and metabolic disorders, so serum UA concentration is considered a prognostic marker in patients with metabolic syndrome [8]. It has also been reported that hyperuricemia may contribute to the development and progression of CVD, T2DM, and metabolic fatty liver by promoting oxidative stress and inflammation [9–11]. Previous reports have also described an association between low or high levels of serum UA and COVID-19 outcomes. However, the precise relationship between COVID-19 severity and serum UA levels is unknown [12–15].

Dyslipidemia as an integral part of metabolic syndrome also seems to be associated with an increased risk of severe COVID-19 infection [6]. Patients with dyslipidemia have high levels of low-density lipoprotein-cholesterol (LDL-c), which leads to the activation of inflammation and causes the release of pro-inflammatory cytokines. In COVID-19 infection, high levels of pro-inflammatory cytokines are associated with severe consequences through a cytokine storm. Also, patients with dyslipidemia have low high-density lipoprotein cholesterol (HDL-c) levels. In addition to removing excess cholesterol from the body, HDL-c has an anti-inflammatory and antioxidant role. Overall, low serum levels of HDL-c can be linked to worse metabolic status. Thus, low serum levels of HDL-c may potentially be a factor that worsens the prognosis of COVID-19 [16–19].

However, some studies have demonstrated that UA to HDL-c ratio (UHR), a combination of two biochemical metabolic parameters is which is a more useful predictor of metabolic disorders including metabolic syndrome, T2DM, and non-alcoholic fatty liver disease (NAFLD) [20–22]. Considering the crucial role of metabolic comorbidities in the occurrence of the severe form of COVID-19, it is obvious that the early estimation of metabolic health using an inexpensive and available biochemical marker such as the UHR index can be very helpful for the assessment of disease prognosis and management of the patients. Therefore, given the limited information regarding the correlation between UHR index and COVID-19 outcomes [23], our study aims to investigate the relationship between serum UA levels and UHR index with the severity and mortality of COVID-19 infection.

## Materials and methods

We conducted this cross-sectional study on 424 non-vaccinated patients admitted to Shahroud University of Medical Sciences (SHMU) Hospitals with a diagnosis of SARS-CoV-2 infection between February 20, 2020, and March 20, 2021. The COVID-19 diagnosis was confirmed by real-time reverse transcriptase-polymerase chain reaction (RT-PCR) from oro- and nasopharyngeal swab specimens. This research was approved by the Ethics Committee of SHMU (IR.SHMU.REC.1400.208) and was performed in line with the principles of the Declaration of Helsinki. Informed consent was received from all the participants before registration in the study.

### Clinical and laboratory assessments

As previously described [6], all clinical and laboratory items of the enrolled patients were extracted from electronic medical records. The electronic medical records were reviewed and SARS-CoV-2 infected cases who underwent UA analysis on admission were included. The cases with incomplete medical records, subjects with known diagnoses of gout, and cases prescribed drugs that will affect UA levels were excluded.

For laboratory assessment, blood samples were drawn from participants in the fasting state (10–12 h fasting) on the first morning following admission. Biochemical parameters such as lipid profile (TC, TG, LDL-C, and HDL-C) and UA were measured using calorimetric methods via commercially available kits (Pars Azmoon, Tehran, Iran). UHR was calculated as serum UA levels (in mg/dL) divided by HDL-C levels (in mg/dL). Weight and height were assessed to calculate BMI as weight in kilograms divided by height in meters squared. The covid-19 severity was defined according to any of the following criteria:1. respiratory rate > 30/min, 2. oxygen saturation ≤ 93%, 3. Patients with shock, or respiratory failure requiring mechanical ventilation, or with other organ failure admission to intensive care unit (ICU). The deceased cases were also defined as severe SARS-COV-2 infected cases.

### Statistical analysis

The chi-squared test and Student’s t-test were utilized to compare the variations between two groups, for qualitative and quantitative variables, respectively. Multivariate logistic regression (adjusted for age, and gender) with severity and mortality of COVID-19 as dependent variable and biochemical parameters as independent variables were conducted. The UHR and HDL-c are grouped based on the cumulative percentage of patients and the amount of these parameters. Patients were divided into three tertiles, with approximately 33% of patients in each group to ensure that groups were evenly distributed. Regarding the UA levels, patients were divided into three groups based on reference range.

The cut-off values for the parameters were selected based on receiver operating characteristic (ROC) curves with the highest sensitivity and specificity.

The statistical analysis was carried out using SPSS software version 23. Any differences with *p*-values less than 0.05 were considered statistically significant.

## Results

The mean age of the 424 confirmed COVID-19 patients in the current study was 59.00 ± 15.62 years old. 202 (47.6%) of cases were males, 92 (21.7) of subjects had a severe form of the disease, and 37 (8.7%) of patients died. Demographic, clinical, and laboratory findings are summarized in Table 1,according to the severity and mortality. Patients with severe disease and cases who died were older, these subjects had a higher proportion of men and a greater history of comorbidities. The comorbidities evaluated in this study included diabetes, CVD, kidney and liver disease, respiratory disorders, cancers, neurological diseases, and seizures (Details have been provided in the previous research [6].

The mean UA and UHR values of the severe group and deceased group were statistically higher than those mild group and survivor group, respectively (*p* < 0.05).

Mild cases exhibited statistically significant higher values of total cholesterol (TC) and LDL-c when compared with severe cases (*p* < 0.05). The mean triglyceride (TG) and HDL-c serum concentrations were not significantly different in the mild and severe groups (*p* > 0.05).

No statistically significant differences could be noted for LDL-c, TC, and TG serum concentration significantly between survivor and deceased groups (*p* > 0.05). Compared to the survivor cases, deceased subjects had lower serum concentrations of HDL-c (*p* < 0.05).

**Table 1:** Clinical, demographic, and Laboratory findings of COVID-19 patients

Variables	Total	Mild (*n* = 332)	Severe (*n* = 92)	*P*-value	Survivor (*n* = 387)	Deceased (*n* = 37)	*P*-value
Gender Male	202 (47.6)	153(46.7)	49(53.3)	0.199	179(46.3)	23(62.2)	**0.046**
Female	222 (52.4)	179(53.8)	43(46.2)		208(53.7)	14(37.8)	
Age (year)	59.00 ± 15.62	56.77 ± 15.36	67.11 ± 13.47	**0.001**	57.97 ± 15.54	69.78 ± 11.93	**0.001**
Comorbidities	222 (52.4)	160 (48.2)	62 (67.4)	**0.001**	198 (51.2)	24 (65.9)	0.071
BMI (kg/m^2^)	28.13 ± 4.32	28.12 ± 4.18	28.15 ± 4.83	0.957	28.19 ± 4.35	27.46 ± 3.93	0.320
UA (mg/dl)	5.0 ± 1.94	4.81 ± 1.73	5.86 ± 2.66	**0.001**	4.93 ± 1.85	6.12 ± 3.05	**0.024**
HDL-C (mg/dl)	32.58 ± 9.75	32.88 ± 9.87	31.00 ± 9.76	0. 104	32.79 ± 9.89	29.13 ± 9.11	**0.023**
UHR	0.17 ± 0.098	0.16 ± 0.11	0.22 ± 0.17	**0.005**	0.17 ± 0.19	0.25 ± 0.23	**0.033**
TG (mg/dl)	105.96 ± 59.28	104.64 ± 60.34	112.1 ± 55.37	0.264	105.22 ± 59.55	116.89 ± 56.46	0.232
TC (mg/dl)	131.13 ± 35.16	132.80 ± 35.14	123.81 ± 35.41	**0.034**	131.33 ± 35.68	125.81 ± 31.81	0.325
LDL-C (mg/dl)	73.57 ± 22.43	74.88 ± 22.21	68.27 ± 22.79	**0.014**	73.74 ± 22.57	70.32 ± 21.50	0.356

Data were expressed as mean ± standard deviation or number (percent)

Abbreviations: BMI, body mass index; UA, uric acid; HDL-c, High-density lipoprotein cholesterol; UHR, UA to HDL-c ratio; TG, Triglyceride; TC, Total cholesterol; LDL-C, Low-density lipoprotein cholesterol

Multivariate logistic regression analysis revealed that predictors of severity and mortality adjusted for age and gender were UA, UHR index, and TG (Table 2). In addition, the prediction for severity and mortality was also determined by the Area Under the Curve (AUC). UA and UHR index could significantly predict the odds of severity and mortality more than lipid profile. The UHR is the best predictor of disease severity (AUC: 0.706) and mortality (AUC: 0.753) compared to UA and HDL-c. As the level of UA rises, the odds of experiencing severe illness and death increase by approximately 20%. However, when the UHR ratio increases by one unit, the odds increase by approximately tenfold. The association of HDL with severity of disease and death was not significant (Table 2).

**Table 2:** The odds ratios for severe and deceased cases associated with uric acid, UHR, lipid profile

	Severity			Mortality		
Parameters	OR(CI)	*P*-value	AUC*	OR(CI)	*p*-value	AUC*
**UA (mg/dl)**	1.20(1.07–1.35)	**0.002**	0.689	1.19 (1.023–1.381)	**0.024**	0.745
**UHR**	10.04(1.50–67.30)	**0.018**	0.706	10.73(1.47.78.11)	**0.019**	0.753
**HDL-C (mg/dL)**	0.981(0.956–1.007)	0.154	0.699	0.963(0.926–1.001)	0.059	0.746
**LDL-C (mg/dL)**	0.989(0.978–1.001)	0.067	0.693	0.999(0.984–1.015)	0.415	0.742
**TC (mg/dL)**	0.995(0.988–1.002)	0.181	0.706	1.00(0.99–1.01)	0.973	0.763
**TG (mg/dL)**	1.004(1.000-1.008)	**0.041**	0.693	1.006(1.001–1.011)	**0.022**	0.752

Regression models adjusted for age and gender

Abbreviations: UA, uric acid; HDL-c, High-density lipoprotein cholesterol; UHR, UA to HDL-c ratio; TG, Triglyceride; TC, Total cholesterol; LDL-C, Low-density lipoprotein cholesterol; AUC, Area Under Curve

According to a logistic model adjusted to age and gender, having UA levels above 7.3 mg/dl compared to the level of 3.6–7.2 mg/dl increases the odds of severe disease and death by 3.5 and 3.36 times, respectively. Additionally, compared to the central tertile, a UHR greater than 0.185, increases the odds of severe disease and death by 2.56 times and 2.71 times, respectively (Table 3). Patients who have HDL-c levels less than 27 mg/dl were at higher risk of severe disease when compared to the levels of 28–35 mg/dl. The low levels of HDL-c had no significant effects on mortality of COVID-19.

**Table 3:** The relationship between serum UA, UHR, and HDL-c with COVID-19 outcomes by categories at admission

Parameters	Group	Total patient	Event Severity			Mortality	
		(number)	(%)	OR(CI)	*P*-value	OR(CI)	*P*-value
**UA (mg/dl)**	(≤ 3.5)	103	18.4	1.29 (0.70–2.39)	0.42	1.36 (0.53–3.46)	0.518
	(3.6–7.2)	278	18.7	1		1	
	(≥ 7.3)	43	48.8	3.49 (1.71–7.12)	**0.001**	3.36 (1.36–8.32)	**0.009**
**HDL tertile** **(mg/dl)**	(≤ 27)	135	28.1	2.18 (1.18–4.02)	**0.013**	1.51 (0.64–3.60)	0.347
	(28–35)	145	15.2	1		1	
	≥ 35	144	34.8	1.51 (0.8–2.85)	0.197	1.12 (0.45–2.80)	0.80
**UHR tertile**	(≤ 0.119)	140	17.1	1.39 (0.72–2.68)	0.328	1.56 (0.57–4.32)	0.38
	2 (0.12–0.184)	142	16.2	1		1	
	3 (≥ 0.185)	142	31.7	2.56 (1.41–4.65)	**0.002**	2.71 (1.12–6.55)	**0.026**

Regression models adjusted for age and gender

Abbreviations: UA, uric acid; HDL-c, High-density lipoprotein cholesterol; UHR, UA to HDL-c ratio

**Fig. 1:**
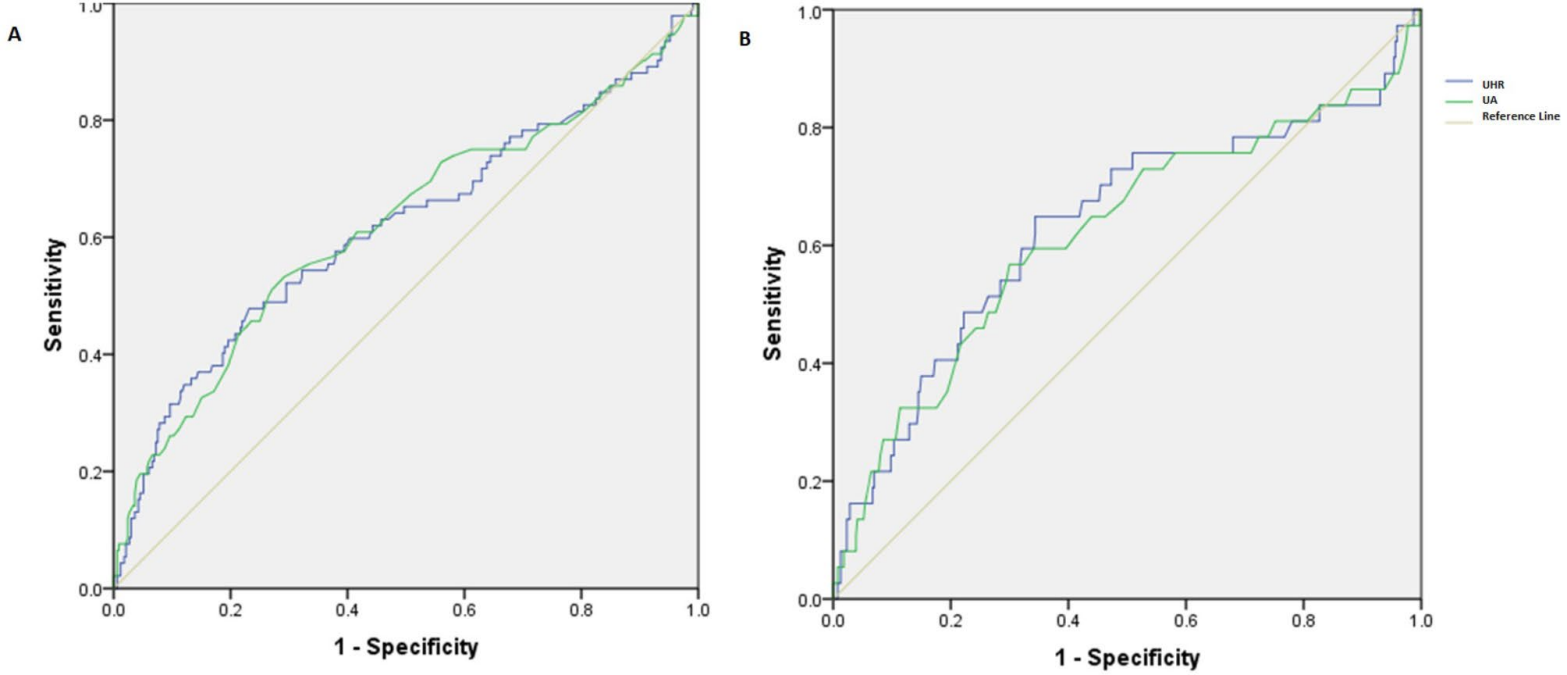
The results of ROC curve analysis regarding the predictability of UA and UHR for severity (**A**), and mortality (**B**) in COVID-19 patients

Figure 1 shows the ROC curve for UA and UHR index as predictors for severity and mortality in COVID-19 patients. The optimal cut-off points of UA and UHR for predicting COVID-19 severity were 4.95 (sensitivity of 61%, specificity of 59%), and 0.151 (sensitivity of 63%, specificity of 53%) (Table 4). The best cut-off value of the UA to predict COVID-19 mortality was 5.05 (sensitivity of 62%, specificity of 59%) and 0.165 (sensitivity of 68%, specificity of 57%), respectively.

**Table 4:** The AUC, sensitivity, specificity by the optimized cut-off points for UA and UHR in predicting the severity and mortality of COVID-19 patients

Outcomes	Parameters	Cut-off	Sensitivity	Specificity
**Severity**	UA	≥ 4.95	0.61	0.59
	UHR	≥ 0.151	0.63	0.53
**Mortality**	UA	≥ 5.05	0.62	0.59
	UHR	≥ 0.163	0.68	0.57

## Discussion

This study was conducted to investigate the relationship between serum UA level and UHR values with severity and mortality of disease in hospitalized COVID-19 patients in the population covered by SHMU. In the present study, it was found that there is a positive and significant relationship between the serum level of UA and UHR index with the severity (OR = 1.20 CI:1.07–1.35, OR = 1.19 CI:1.023–1.381 respectively) and mortality (OR = 10.04 CI:1.50–67.30, OR = 10.73 CI:1.47–87.11) of COVID-19.

Serum UA is known as the end product of purine degeneration. Although UA is identified as an antioxidant and immune system mediator, it has been revealed that hyperuricemia has pathophysiological roles in oxidative stress, inflammation, and insulin resistance [24]. Studies demonstrated that serum UA level increases in respiratory diseases and hypoxic conditions [4, 25]. Besides, it is possible that high levels of serum UA and a high ratio of uric acid to creatinine have a significant relationship with functional and clinical characteristics of patients with acute exacerbation of chronic obstructive pulmonary disease [12]. In Chauhan et al.‘s study, they stated that serum level of UA can be used as a predictive biomarker for the mortality and the severity of COVID-19, in such a manner that higher levels of UA can increase the severity of the disease and expand the hospitalization period. In addition, high serum UA is associated with a higher probability of recurrence [26]. In accordance with the mentioned study, our results also showed a statistically significant relationship between the serum UA level and the severity and mortality of patients. Chen et al., revealed that the relationship between serum level of UA and critical outcomes of COVID-19 is U-shaped, suggesting that compared to the baseline serum UA (279 to 422 µmol/L) both higher and lower UA concentrations are risk factors for poor outcomes in COVID-19 patients [27]. Although in the present study, compared to the level of 3.6–7.2 mg/dl, hypouricemia also showed a weak association with increased risk of severity and mortality of COVID-19 patients, but this relationship was not significant. The smaller sample size in our study may be the reason for this discrepancy.

According to the results of several studies, dyslipidemia can be considered as one of the risk factors of severe infection of COVID-19 [16] especially through activation of inflammation, release of pro-inflammatory cytokines, and disruption of the regulation of innate immune response [16, 18, 28]. However, it is worth noting that in the present study and our previous study [6], we revealed that among the various lipid profile items, only triglyceride was a risk factor for disease severity and mortality, after removing the effect of confounding factors. Also, the present study revealed that patients who have HDL-c levels less than 27 mg/dl increased the risk of severe disease almost 2fold when compared to the levels of 28–35 mg/dl.

Data coming from various researches demonstrate that loss of metabolic health that is associated with different comorbidities is an important risk factor for severe COVID-19. Therefore, studies showed that using simple and inexpensive metabolic indices in order to assess of metabolic status of COVID-19 patients could be useful for determining COVID-19 prognosis [6]. In the previous study, we showed that several metabolic indices calculated from biochemical parameters including lipid ratios and TyG index were better predictors for disease severity and mortality than lipid profile alone in COVID-19 patients [6]. In the present study, we also suggested that the UHR, as a good combined biochemical marker calculated from serum levels of UA and HDL-c has a significant and direct relationship with the severity and mortality of COVID-19. Also, we revealed that compared to the central tertile, a UHR greater than 0.185, increases the odds of severe disease and death by 2.56 times and 2.71 times, respectively.

It has been demonstrated that UHR can be a promising indicator for the diagnosis and treatment of patients with metabolic disorders including metabolic syndrome, T2DM, NAFLD, and inflammatory disorders [14, 28]. Aktas et al. observed that UHR has a direct relationship with glucose and HbA1C and can be used as a predictive marker of diabetes in males [29]. Also, in another study that was conducted with the aim of investigating the HUR index and incident ischemic heart disease (IHD) in the population of non-diabetic Koreans, it was found that increased values of UHR had a positive relationship with the occurrence of IHD and its increase is a useful measure to assess the risk of cardiovascular disease in the preclinical stage [30]. Furthermore, a positive clinical correlation between increased UHR and NAFLD disease has been observed in the study of Hui et al., in a Chinese adult population and UHR has been introduced as an innovative and non-invasive marker to identify people at risk of NAFLD [31]. Besides, the very same relationship was observed in Yazdi et al’s cross-sectional study, which proved UHR to be a helpful marker for diagnosing and screening people at risk of metabolic syndrome [8]. Here in our study, for the first time, we found that UHR in those who died due to coronavirus was significantly higher than recovered ones, and the higher this ratio is, the severity of the disease would increase, which indicates the importance of this index in predicting the severity and mortality rate of the disease.

## Conclusion

In conclusion, our results showed that the UHR index is more powerful for predicting the severity and mortality of covid-19 than UA or lipid profile alone. Therefore, the calculation of the UHR index in the initial days of hospitalization of patients can be useful for predicting the prognosis of the disease and can help to follow up the treatment of COVID-19 patients.

### Limitation

This study is the first study regarding the correlation between the UHR and critical outcomes of COVID-19. However, this study has several limitations. First, the sample size in our study was small because the serum UA measurement was not routinely performed in all COVID-19 hospitalized patients. Second, it was not possible to ignore SARS-COV-2 impact on the UA and UHR at the early stage of COVID-19 diagnosis because of the lack of biochemical examination before infection. Third, since we utilized the primarily accessible serum UA measure, the timing of serum UA estimation was not uniform in all COVID-19 cases.

## Data Availability

The datasets generated and/or analysed during the current study are not publicly available due to reasons of sensitivity to protect study participant privacy but are available from the corresponding author on reasonable request.
